# Development of an infant feeding core outcome set for childhood obesity interventions: study protocol

**DOI:** 10.1186/s13063-017-2180-4

**Published:** 2017-10-10

**Authors:** Karen Matvienko-Sikar, Molly Byrne, Colette Kelly, Elaine Toomey, Marita Hennessy, Declan Devane, Caroline Heary, Janas Harrington, Niamh McGrath, Michelle Queally, Patricia M. Kearney

**Affiliations:** 1 School of Public Health, University College Cork, Cork, Ireland; 2 School of Psychology, National University of Ireland Galway, Galway, Ireland; 3 Discipline of Health Promotion, School of Health Sciences, National University of Ireland Galway, Galway, Ireland; 4 School of Nursing and Midwifery, National University of Ireland Galway, Galway, Ireland; 5 Discipline of Economics, National University of Ireland Galway, Galway, Ireland

**Keywords:** Core outcome set, Infant feeding, Childhood obesity

## Abstract

**Background:**

Childhood obesity is a significant public health challenge that affects approximately one in five children worldwide. Infant feeding practices are implicated in the aetiology of childhood obesity. Infant feeding interventions for childhood obesity are increasingly popular but outcome reporting is inconsistent across trials. Lack of standardisation limits examination of intervention effects and mechanisms of change. The aim of the current project is to develop a core set of infant feeding outcomes for children ≤ 1 year old, to be evaluated in childhood obesity intervention trials.

**Methods:**

This project will use similar methodology to previous core outcome development research. An infant feeding core outcome set (COS) will be developed in four stages: (1) a systematic review of the literature, (2) discussion and clarification of outcomes in a meeting involving multiple stakeholder perspectives, (3) prioritisation of outcomes using the Delphi technique with an expert panel of stakeholders, and (4) achieving consensus on the COS using the nominal group technique (NGT) consensus meeting. An online Delphi survey will be conducted following the NGT meeting to prioritise outcomes identified in the systematic review. An NGT meeting will be conducted with groups of health professionals, non-clinician researchers, and parents of infants ≤ 1 year old, to achieve final consensus on the infant feeding COS.

**Discussion:**

This study aims to develop a core outcome set of infant feeding outcomes for randomised infant feeding studies to prevent childhood obesity. This research will improve examination and syntheses of the outcomes of such studies to prevent and reduce childhood obesity.

**Electronic supplementary material:**

The online version of this article (doi:10.1186/s13063-017-2180-4) contains supplementary material, which is available to authorized users.

## Background

Childhood obesity is a global public health challenge [1]. Rates of childhood obesity have nearly doubled since 1980 [1]. Currently approximately 1 in 5 children worldwide are classified as overweight or obese [2]. Being overweight or obese in childhood are strong predictors of adverse health and wellbeing outcomes in childhood and later life. These include diabetes mellitus [3], cardiovascular disease and a range of cancers [4], depression and low health-related quality of life [5]. Obese children are also more likely to become obese adults [6].

Parents’ early feeding practices contribute to the aetiology of becoming overweight or obese [7]. These practices contribute to childhood weight outcomes via the duration of breastfeeding and timing of introduction to solids, dietary intake and quality of foods provided [7], and parent-child feeding interactions [8]. Parental behaviours are also suggested to influence child eating behaviours [9], which track to adulthood [10]. Parents’ early feeding practices, particularly in the first year, are therefore important modifiable behaviours for reducing children’s risk of being overweight or obese.

Childhood obesity prevention interventions increasingly target feeding practices in early infancy [11]. To date infant feeding interventions have demonstrated inconsistent findings for child weight outcomes [11]. It is assumed in the extant literature that the effects of infant feeding interventions on weight outcomes operate via the mechanism of improved infant feeding. However, a recent systematic review of the effects of infant feeding interventions on feeding outcomes highlighted pervasive heterogeneity in outcomes examined and reported (Matvienko-Sikar et al.: Effects of early infant feeding interventions on parental feeding practices: a systematic review, submitted). Evaluation and examination of the mechanisms of change underpinning interventions is therefore hampered by a lack of consistency and standardisation of infant feeding outcomes.

Development of a core outcome set (COS) of infant feeding outcomes is urgently needed. A COS, or standardised set of outcomes, represents the minimum that should be measured and reported in all trials for a specific clinical area of health or health care [12]. A COS enhances the value of evidence syntheses by reducing outcome heterogeneity and risk of outcome reporting bias, because trial findings include a presentation of these core outcomes at a minimum [12]. Developing a COS of infant feeding outcomes is therefore essential for the development and evaluation of randomised studies of infant feeding interventions for childhood obesity.

### Objective

The aim of this study is to develop an infant feeding COS that can be used in randomized controlled studies of infant feeding interventions to prevent childhood obesity, which include infants < 1 year old. This will be done based on a systematic review of the peer-reviewed extant literature; a meeting involving multiple stakeholder perspectives to discuss and clarify outcomes; an e-Delphi survey involving an expert panel of international stakeholders; and a nominal group technique (NGT) consensus meeting to finalise the COS.

The objectives of this study are:

1. To identify all potential infant-feeding outcomes for infants up to 1 year of age in the extant literature
2. To achieve consensus on a COS for infant feeding of infants up to 1 year of age using the Delphi and nominal group techniques

## Methods

The development of this COS adheres to the recommendations provided by the Core Outcome Measures in Effectiveness Trials (COMET) initiative and the Core Outcome Set-Standards for Reporting (COS-STAR) statement [13].

**Fig. 1:**
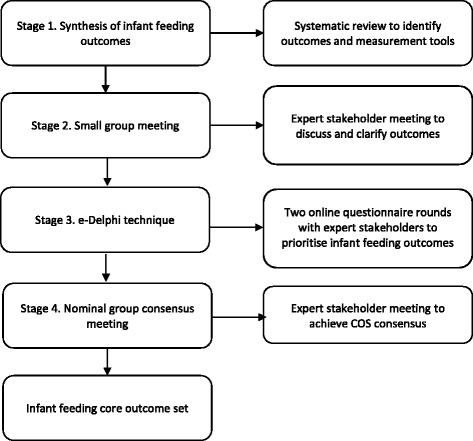
Overview of core outcome set (COS) development

This study will proceed in 4 distinct phases (see Fig. 1):

1. Synthesis of existing outcomes to generate a long-list of potential outcomes and measurement tools
2. Discussion and clarification of outcomes in a meeting involving multiple stakeholder perspectives
3. Prioritisation of outcomes using the e-Delphi technique with an expert panel of stakeholders
4. Achieving consensus on the COS using the NGT consensus meeting with an expert panel of stakeholders

### Stage 1: synthesis of existing outcomes to generate a long-list of potential outcomes

A systematic literature search will be conducted to identify all potential infant-feeding outcomes and outcome measurement instruments in the extant literature.

#### Search strategy

The search will involve a systematic review of infant feeding outcomes using electronic databases including EMBASE, MEDLINE, CINAHL, the Cochrane Library, and PsychINFO. Reference lists of relevant articles will also be examined. There will be no language or publication date restrictions but the search will be limited to published literature. Outcomes will be identified from studies describing randomised controlled trials, quasi-experimental, observational, pilot, and feasibility studies that examine infant feeding outcomes. This search strategy acknowledges that infant feeding outcomes suitable for use in trials of obesity-prevention interventions may also arise from existing observational studies. Two recent qualitative evidence syntheses (QES) of parents’ experiences of infant feeding (Matvienko-Sikar K: Parental experiences and perceptions of infant complementary feeding behaviours: a qualitative evidence synthesis, in preparation; Toomey Eea: Healthcare professional and parental views and experiences of participating in infant feeding interventions: aqualitative evidence synthesis, in preparation) will also be examined to identify parent-reported outcomes of importance. Conducting an additional QES specifically looking at outcomes was deemed inappropriate and potentially representative of research waste in light of these recent syntheses.

#### Inclusion criteria

Studies were included that had examined at least one infant-feeding outcome in children < 1 year of age. Studies examining outcomes in children > 1 year of age will be excluded. Studies examining breastfeeding only were excluded﻿. Studies involving children with malnutrition, ongoing medical conditions related to feeding, or studies focusing on dental caries will be excluded. There will be no restrictions on child sex or ethnicity.

#### Eligibility of studies

Titles and abstracts of studies retrieved will be independently screened against inclusion criteria by two members of the research team. The full text of all potentially eligible studies will then be independently assessed for eligibility by one researcher. One third of all full texts will also be randomly selected and checked by a second reviewer. Any disagreements will be resolved through consensus, with the involvement of a third reviewer if necessary; if this cannot be achieved, disagreements will be resolved through discussion with a third reviewer.

#### Data extraction

A standardised, data extraction form will be used to extract the following data: author, year of publication, study design, sample size, study setting, participant characteristics, infant feeding outcomes, and outcome measurement tool. Study quality will be measured using six items previously outlined in a core outcome set for neonatal abstinence syndrome [14]. This includes items examining clarity of outcome presentation, definition, measurement, and explanation of outcome selection.

#### Data analysis

Characteristics of studies and study quality will be presented descriptively and/or in tables. Studies will be grouped to form domains based on outcomes examined. Studies will be grouped to form domains based on outcomes examined. This will involve examination of individual outcomes reported across studies to identify thematically similar outcomes that can be grouped and represented under overarching categories or domains. It is expected for instance, that an outcome domain of “breastfeeding” may contain various outcomes including initiation, duration, and frequency. Study outcomes will be narratively synthesised to develop a long-list of infant-feeding outcomes and associated measurement instruments.

### Stage 2: discussion and clarification of outcomes in a meeting involving multiple stakeholder perspectives

Following identification of the long-list of infant-feeding outcomes, a group meeting will be conducted with a group of expert stakeholders to discuss and clarify identified outcomes and outcome domains.

#### Participants

This meeting will involve a group of 10–15 expert national and international stakeholders from the following stakeholder groups:

- Paediatricians
- Dieticians
- Nutritionists
- General Practitioners
- Public Health Nurses
- Practice Nurses
- Childcare Providers
- Non-clinician researchers in the area of infant feeding and childhood obesity

Healthcare professionals will be recruited from national healthcare practices and national and international organisations (e.g. Irish Society for Clinical Nutrition and Metabolism; Association for Nutrition). Non-clinician researchers will be identified from international research organisations and networks (e.g. The British Feeding and Drinking Group; Health Research Board Trials Methodology Research Network). Childcare providers will be identified from City/County Childcare Committees (CCC) and Voluntary Childcare Organisations (VCOs). This will facilitate inclusion of a range of views while maintaining a good group dynamic.

#### Procedures

Prior to commencement of the meeting, the list of outcomes identified in stage 1 will be summarised in plain language suitable for members of all stakeholder groups. The plain language summaries on COSs compiled by the COMET initiative will also be provided to participants. Outcomes within each identified domain will be presented to participants for discussion, which will be conducted in a neutral manner. Participants will engage in a ranking exercise to provide information on perceptions of outcome importance within the group. Following presentation and discussion of outcomes participants will rate each outcome from 1 to 9. The ranking outcomes of the first rating exercise will then be presented back to participants to be re-rated, as previously, from 1 to 9. The aim of the meeting is not to prioritise or eliminate any outcomes that will be brought forward to the e-Delphi however. It is intended instead to allow for discussion and clarity on proposed outcomes, as informed by expert stakeholders.

### Stage 3: prioritisation of outcomes using the Delphi technique with an expert panel of stakeholders

Following stage 2, an e-Delphi exercise will be conducted with an international group of expert stakeholders. The Delphi technique has been previously used in the development of COSs [15]. It involves a panel of participants with expertise in the given area, individually completing a series of sequential questionnaires. After each questionnaire, group responses are tabulated and reported back to the group who are asked to complete a subsequent questionnaire [15]. This process facilitates achieving a consensus on outcomes of importance through iterative evaluation and condensing of participant responses. e-Delphi exercises allow this process to be conducted online and have been previously used in COS development [16]. The use of e-Delphi surveys facilitates international participation with increased cost and time efficiency, and reduced risk of attrition, as compared to traditional postal surveys [16]. In the current study, an e-Delphi technique will be used to obtain input from an international group of expert stakeholders and achieve consensus on outcomes for the COS. This will consist of three rounds of sequential questionnaires administered online to the panel of stakeholders.

#### Participants

As there are no generally accepted guidelines for Delphi panel size, this project will recruit as large a panel as possible with a proposed minimum of 20 participants per stakeholder group. This has been chosen in order to account for potential attrition and to increase diversity of responses within and across stakeholder groups. Nine stakeholder groups will be involved in the Delphi study; a minimum of 180 participants will therefore be recruited. The stakeholder groups involved in the Delphi study are the same as those identified for stage 2 but will also include parents of infants up to 1 year of age. As in stage 2, healthcare professionals will be recruited from national healthcare practices and though national and international professional societies and associations (e.g. Irish Society for Clinical Nutrition and Metabolism, European Paediatric Association). Non-clinician researchers will be identified from international research organisations and networks (e.g. The British Feeding and Drinking Group; Health Research Board Trials Methodology Research Network). Corresponding authors of relevant publications retrieved in the systematic literature search (stage 1), will also be contacted by email to participate. Childcare providers will be identified from CCCs and VCOs. Parents of infants up to 1 year of age will also be included in the Delphi survey. Parents will be recruited from online parenting forums (e.g. www.babycentre.com, www.eumom.ie) by posting study information and contact details on the online forum.

All participants will be informed of the purpose of the study, their right to withdraw at any time, and that all data provided will be kept confidential and anonymised. Participants will provide consent to participate on the initial online questionnaire.

#### Procedures

Prior to commencement of the Delphi exercise, the list of outcomes identified in stage 1 will be summarised in plain language suitable for members of all stakeholder groups. The plain language summaries on COSs and the Delphi technique compiled by the COMET initiative will also be provided to participants. Participants will be asked to provide information on their education, employment, and experience with infant feeding (in a professional or parental role, dependent on stakeholder group).

##### Round 1

In round 1 of the Delphi exercise, the initial list of outcomes will be circulated to participants in all stakeholder groups. Participants will be given 2 weeks to complete the initial questionnaire. Utilising the scale proposed by the Grading of Recommendations Assessment, Development and Evaluation (GRADE) working group, and as discussed by Williamson et al. [17], participants will be asked to rate the importance of proposed outcomes between 1 and 9. A score of 1 to 3 represents limited importance; a score of 4 to 6 indicates the outcome is important but is not critical; a score of 7 to 9 represents a critical outcome. Participants will also be provided an opportunity to suggest up to five additional outcomes that they feel warrant inclusion.

##### Analysis round 1

Responses from round 1 will be collated and scores for each outcome will be calculated as a percentage of the total responses for all scores. Scores for the parent subgroup will also be examined separately and compared descriptively to the full group scores to ensure their voices are being heard.

##### Round 2

In round 2, participants will be shown their own scores from round 1, and the distribution of scores for each outcome per stakeholder group. Participants will be asked to rate the outcomes again following the same format as round 1, using the scale of 1 to 9. Any new outcomes identified in round 1 will also be included in the round 2 questionnaire. As in round 1, participants will be requested to complete the survey within 2 weeks.

##### Analysis round 2

Following round 2, scores will again be tabulated. Outcomes that 70% of participants rated from 7 to 9 and fewer than 15% of participants rated as 1 to 3 [12] will be considered critical for inclusion. Conversely, outcomes that 70% or more of participants rated as 1 to 3 and fewer than 15% rated as 7 to 9 [12] will be considered not important for inclusion in the COS and will not be brought forward to round 3.

##### Round 3

In round 3, participants are shown their own scores from the previous round, and the distribution of scores for each outcome per stakeholder group. Rating will follow the same format as rounds 1 and 2, using the scale of 1 to 9. Participants will be requested to complete the survey within 2 weeks.

##### Analysis round 3

Following round 3, scores will be tabulated. Consensus on inclusion of an outcome will be defined as 70% or more of participants scoring an outcome from 7 to 9 and fewer than 15% of participants scoring it as 1 to 3 [12]. Conversely, consensus on exclusion of an outcome will be defined as 70% or more participants scoring the outcome as 1 to 3 and fewer than 15% scoring it as 7 to 9 [12]. Outcomes that achieve consensus for inclusion, and those that do not meet consensus for exclusion will be brought forward to stage 4.

### Stage 4: achieving consensus on the COS using the NGT consensus meeting with an expert panel of stakeholders

Following the e-Delphi survey, an NGT consensus meeting will be conducted with a group of national and international expert stakeholders. Inclusion of a face-to-face consensus meeting following a Delphi survey is recommended by the COMET initiative [18]. This is because the aim of the Delphi survey is to prioritise and identify core outcomes, rather than achieving consensus for every outcome. A face-to-face meeting enables final consensus to be reached by a representative group of expert stakeholders on all outcomes.

#### Participants

The NGT is a small group approach to achieving consensus. It will involve a group of 10–15 expert national and international stakeholders from the following stakeholder groups:

- Paediatricians
- Dieticians
- Nutritionists
- General Practitioners
- Public Health Nurses
- Practice Nurses
- Childcare Providers
- Non-clinician researchers in the area of infant feeding and childhood obesity

Participants for the NGT meeting will be recruited in a similar manner to that outlined in stage 2.

#### Procedures

The NGT will follow standard procedures. Prior to commencement of the NGT exercise, the list of outcomes identified in stage 3 will be summarised in plain language suitable for members of all stakeholder groups. The plain language summaries on COSs compiled by the COMET initiative will also be provided to participants. Outcomes from the e-Delphi study will be presented to participants and information will be provided regarding whether the e-Delphi participants reached consensus on the outcome or not. Participants will discuss and share ideas as a group about the outcomes for inclusion in the COS. Group discussion of ideas from each group will be conducted in a neutral manner, without the elimination of any outcomes. Following presentation and discussion of outcomes participants will rate each outcome for inclusion (“yes” or “no”). Outcomes that 70% or more of participants rated as “yes” for inclusion will then be discussed, prior to a final rating of outcomes for inclusion in the COS. Outcomes that 70% or more of participants rated as “yes”’ for inclusion in the final ranking will be included in the final COS.

### Ethical arrangements

Ethical approval for this study has been obtained from the Social Research Ethics Committee of University College Cork. Ref. 2016-211.

## Discussion

There is currently no infant-feeding COS that can be used in randomised controlled trials of childhood obesity prevention interventions. Inconsistencies and lack of standardisation in current approaches to infant-feeding outcome assessment hinders examination of intervention effects and mechanisms. It further limits evidence syntheses and increases the likelihood of reporting bias.

The present research will standardise examination and reporting of infant-feeding outcomes, thereby improving childhood obesity intervention research. It will propose a core outcome set recommending what outcomes should be measured in randomised trials of infant-feeding intervention involving children up to 1 year old, or at least considered in such trials. The involvement of multiple stakeholder perspectives, through the use of the Delphi and NGT, will ensure applicability and relevance of outcomes within the COS. The objective of this research is therefore to develop an internationally relevant COS of infant-feeding outcomes that is relevant to randomised trials for prevention of childhood obesity (Additional file 1).

### Trial status

Systematic review and meeting with stakeholders is complete; e-Delphi is due for commencement.

## Additional file

Additional file 1: SPIRIT 2013 Checklist: Recommended items to address in a clinical trial protocol and related documents*. (DOC 122 kb)

## Abbreviations

CCC: City/County Childcare Committee
COMET: Core Outcome Measures in Effectiveness Trials
COS: Core outcome set
COS-STAR: Core Outcome Set-Standards for Reporting
GRADE: Grading of Recommendations Assessment, Development and Evaluation
NGT: Nominal group technique
QES: Qualitative evidence synthesis
VCO: Voluntary Childcare Organisation
